# Injectable Antioxidant and Oxygen-Releasing Lignin Composites to Promote Wound Healing

**DOI:** 10.1021/acsami.2c22982

**Published:** 2023-04-06

**Authors:** Swathi Balaji, Walker D. Short, Benjamin W. Padon, Jorge A. Belgodere, Sarah E. Jimenez, Naresh T. Deoli, Anna C. Guidry, Justin C. Green, Tanuj J. Prajapati, Fayiz Farouk, Aditya Kaul, Dongwan Son, Olivia S. Jung, Carlos E. Astete, Myungwoong Kim, Jangwook P. Jung

**Affiliations:** † Division of Pediatric Surgery, Department of Surgery, Texas Children’s Hospital and Baylor College of Medicine, Feigin Center at Texas Children’s Hospital, 1102 Bates Ave, C.450.05, Houston, Texas 77030, United States of America; ‡ Department of Biological Engineering, Louisiana State University, 149 E.B. Doran Hall, Baton Rouge, Louisiana 70803, United States of America; § Louisiana Accelerator Center, University of Louisiana at Lafayette, 20 Cajundome Boulevard, Lafayette, Louisiana 70506, United States of America; ∥ Department of Chemistry and Chemical Engineering, Inha University, Incheon 22212, Republic of Korea

**Keywords:** lignosulfonate, wound healing, reactive oxygen species, calcium peroxide, vascularization

## Abstract

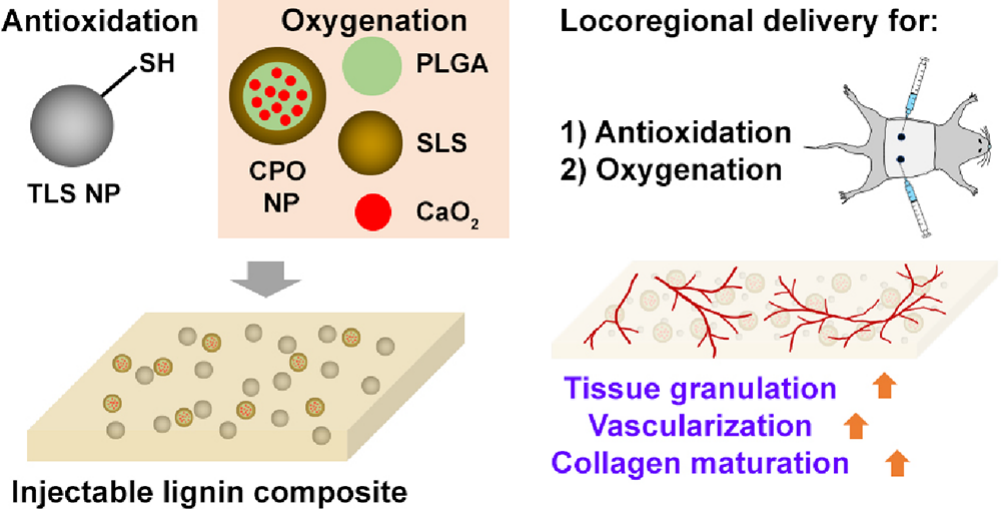

The application of engineered biomaterials for wound healing has been pursued since the beginning of tissue engineering. Here, we attempt to apply functionalized lignin to confer antioxidation to the extracellular microenvironments of wounds and to deliver oxygen from the dissociation of calcium peroxide for enhanced vascularization and healing responses without eliciting inflammatory responses. Elemental analysis showed 17 times higher quantity of calcium in the oxygen-releasing nanoparticles. Lignin composites including the oxygen-generating nanoparticles released around 700 ppm oxygen per day at least for 7 days. By modulating the concentration of the methacrylated gelatin, we were able to maintain the injectability of lignin composite precursors and the stiffness of lignin composites suitable for wound healing after photo-cross-linking. *In situ* formation of lignin composites with the oxygen-releasing nanoparticles enhanced the rate of tissue granulation, the formation of blood vessels, and the infiltration of α-smooth muscle actin^+^ fibroblasts into the wounds over 7 days. At 28 days after surgery, the lignin composite with oxygen-generating nanoparticles remodeled the collagen architecture, resembling the basket-weave pattern of unwounded collagen with minimal scar formation. Thus, our study shows the potential of functionalized lignin for wound-healing applications requiring balanced antioxidation and controlled release of oxygen for enhanced tissue granulation, vascularization, and maturation of collagen.

## 1 Introduction

Wound healing is a critical process that progresses through tightly regulated phases and ultimately leads to the repopulation of the wound with cells and extracellular matrix (ECM) to repair the injured site. A key aspect of the wound-healing process involves the production of granulation tissue, a densely vascularized provisional tissue composed of fibroblasts (FBs), vascular endothelial cells (ECs), inflammatory cells, and cell-derived ECM. Poor vascularization of the granulation tissue is often associated with impaired healing. Recent evidence further links the excessive production of reactive oxygen species (ROS) and/or impaired detoxification of ROS to the pathogenesis of impaired wound healing.^1^ Excess ROS accumulation disrupts cellular homeostasis and causes nonspecific damage to critical cellular components and function, leading to impairment such as abhorrent FB collagen synthesis^2^ and cell apoptosis, EC and smooth muscle cell dysfunction, compromised tissue perfusion, and increased proinflammatory cytokine secretion by macrophages.^3^ Excess ROS is scavenged by enzymes, such as superoxide dismutase and antioxidants, that regulate the redox environment in healing skin wounds.

In recent years, antioxidants have drawn much attention as potential therapeutic interventions due to their ability to fight oxidative stress.^4−6^ The main function of antioxidants is to scavenge or neutralize free radical formation and to inhibit the deleterious downstream effects of ROS. However, most antioxidants, taken orally, have limited absorption profiles, which lead to low bioavailability and insufficient concentrations at the target site.^7^ To overcome this issue, research has focused on developing tissue engineering strategies to provide locoregional delivery of antioxidants. Strategies including antioxidant nanoparticles made of inorganic materials, such as mesoporous silica, cerium oxide, and fullerene, have been evaluated in *in vitro* assays and in animal models to determine their ability to scavenge free radicals while decreasing ROS concentrations to protect cells against oxidative stress.^8,9^ Hydrogels that release ROS scavengers have been developed to promote cell function as a method to mitigate the foreign body response.^10,11^ Additionally, the decellularized myocardial matrix has been shown to protect cardiomyocytes from ROS after myocardial infarction.^12^

Lignin is a polyphenolic polymer that functions in plants to isolate pathogens to the site of infection while providing impermeability to cell walls.^13,14^ We^15^ and others^16^ found that lignosulfonate can form nanostructures, which inspired us to apply lignosulfonate as a nanoscale carrier for drugs or therapeutics while capitalizing on the inherent antioxidant properties of lignosulfonate. The successful application of engineered biomaterials for wound healing relies upon overcoming the limitation of oxygen diffusion via exogenous oxygenation such as using perfusion bioreactors. In small-scale oxygenation, many solid inorganic peroxides have been used to support cell growth, survival, tissue regeneration, and bioremediation.^17^ Calcium peroxide (CaO_2_) possesses many distinctive properties in comparison with other peroxides, including better thermal stability, environmentally harmless end products, extended-release period of hydrogen peroxide, and reasonable cost.^18^ On the basis of the relatively low solubilities (CaO_2_ 1.65 mg/mL at 20 °C and MgO_2_ 0.86 mg/mL at 18 °C^19^), calcium peroxide has a higher oxygen-generation potential than magnesium peroxide.^20^

Therefore, to apply antioxidation and locoregional oxygenation, we developed injectable lignin composites with the following components and properties: (1) lignosulfonate with thiolation (TLS) that scavenges ROS from wounds,^21,22^ (2) unmodified sodium lignosulfonate (SLS) that encapsulates CaO_2_ while scavenging radicals from CaO_2_^15,23^ and simultaneously protecting CaO_2_ from an aqueous environment of tissue or hydrogel, and (3) methacrylated gelatin (GelMA) that modulates the mechanical properties^22^ of lignin composites to support injectability. The first two rationales can confer the dual functionality of lignosulfonate. First, we assessed the integration of CaO_2_ to SLS-PLGA (poly(lactic-*co*-glycolic) acid) nanoparticles (NPs)^15,23^ and the release of O_2_ from lignin composites. Our rationale is to utilize the core–shell structure of SLS-PLGA NPs to deliver CaO_2_ and to protect CaO_2_ from aqueous microenvironments, while NPs, after depleting CaO_2_, still serve as a ROS scavenger. Then, swelling/degradation profiles and mechanical properties of lignin composites were assessed. Last, we applied lignin composites in the wounds of wild-type mice and assessed wound-healing responses including tissue granulation, neovascularization, inflammatory responses, and scarring outcomes.

## 2 Materials and Methods

### 2.1 Dynamic Light Scattering (DLS) of NPs

NPs were synthesized with the previously published methods.^15^ The analysis of SLS-PLGA showed a spherical, core–shell structure with a relatively smooth surface as evidenced by small-angle scattering data and transmission electron micrographs.^23^ The size, polydispersity, and ζ-potential of NPs of CPO and TLS (0.2–0.4 mg/mL) were measured by dynamic light scattering (DLS) using Malvern Zetasizer ZS (Malvern Panalytical, Westborough, MA, USA). NP suspensions were filtered through a 0.45 μm filter. To prevent the formation of disulfide bonds, the TLS sample was prepared in 250 mM tris(2-carboxyethyl) phosphine hydrochloride (TCEP HCl, cat. #H51864, Alfa Aesar, Haverhill, MA, USA) in PBS.

### 2.2 Elemental Analysis of NPs

We utilized the nuclear microscopy setup at the Louisiana Accelerator Center to probe the concentrations of calcium in our samples using particle-induced X-ray emission (PIXE) spectrometry.^24^ The samples were placed in a low-pressure environment (≤1 × 10^–6^ mbar) on an electrically conductive nonporous carbon tape attached to the sample holder. A focused (10 × 10 μm) 2 MeV proton beam, with a beam current in the range of 10–20 pA, raster scanned the sample region (1 × 1 mm) for about 1 h. A silicon drift detector was placed at 135° in front of the sample to detect the characteristic X-rays excited by energetic protons. Analysis of the spectra was performed with the GeoPIXE software (v7.3).^25^ The elemental maps and the derived concentrations were generated by the dynamic analysis method using average matrix composition from the whole scanned area.

### 2.3 Formation of Lignin Composites

TLS was synthesized with the previously published protocol.^21^ GelMA was synthesized by the coupling reaction of gelatin with methacrylic acid (see the Supporting Information for details). We confirmed the alkene incorporation to gelatin with ^1^H nuclear magnetic resonance (NMR, Figure S1). Lignin composites were formed by weighing out GelMA, lithium phenyl-2,4,6-trimethylbenzoylphosphinate (LAP, Allevi, Philadelphia, PA, USA), and NPs of TLS, SLS-PLGA/CaO_2_, or SLS-PLGA (w/o CaO_2_) and by mixing GelMA with LAP and NPs in PBS at 37 °C as summarized in Table 1. Any concentration of TLS higher than 7 mg/mL interfered with the photo-cross-linking of lignin composites, leading to the reduction of elasticity of lignin composites with partially cross-linked lignin composites. The concentrations of GelMA, LAP, and TLS were fixed at 50, 5, and 3 mg/mL, respectively, and the concentrations of SLS-PLGA/CaO_2_ or SLS-PLGA (w/o CaO_2_) were varied at either 4 or 40 mg/mL. Each precursor was pipetted into a custom polydimethylsiloxane (PDMS) mold (8 mm diameter and 1 mm height, 50 μL) to form lignin composites. Samples were cross-linked using a UV flood lamp (Intelli-Ray 400, Uvitron international, West Springfield, MA, USA) for 30 s at 10 mW/cm^2^.

**Table 1:** Abbreviation of Lignin Composites

abbreviation	matrix	nanoparticles (NPs)
UNTX	none	none
GelMA	GelMA	none
TLS	GelMA	TLS
CPOc	GelMA	TLS and SLS-PLGA (w/o CaO_2_)
CPO	GelMA	TLS and SLS-PLGA/CaO_2_

### 2.4 Quantification of O_2_ Release from Lignin Composites

The released O_2_ was optically measured by a planar O_2_ sensor spot (SP-PSt3-SA23-D5-OIW-US, PreSens, Regensburg, Germany) placed at the bottom of a 96-well plate. Lignin composites (5 mm diameter and 1 mm height, 20 μL) were placed on top of the planar O_2_ sensor spot and submerged in serum-free medium (37 °C/5% CO_2_). To prevent evaporation, each well was covered with a sealing film, and the entire plate was wrapped with a sealing tape. A polymer optical fiber (POF-L2.5-2SMA, PreSens, Regensburg, Germany) optically sensed the concentration of O_2_ through the bottom of a well and sent signals to an O_2_ sensor (OXY-1 SMA, PreSens, Regensburg, Germany)^26^ for measurement.

### 2.5 Swelling Ratio and *In Vitro* Degradation of Lignin Composites

Swelling ratios of lignin composites were determined by the following equation: [*W*_s_ – *W*_d_]/*W*_d_, where *W*_s_ and *W*_d_ represent the weight after swelling in PBS for 24 h and the weight after lyophilization, respectively. *In vitro* degradation of lignin composites was determined by submerging lignin composites in a solution of collagenase type II (0.5 U/mL, cat. #CLS-2, Worthington Biochemical) with 1 mM CaCl_2_ in a serum-free culture medium, along with a control group without including the collagenase. Lignin composites were collected at 0, 2, 6, and 24 h and lyophilized to determine the fraction of remaining composites.

### 2.6 Oscillating Rheometry of Lignin Composites

Using a TA Discovery HR-2 rheometer and the previously published methods,^21^ the viscosity of the precursors of lignin composites was measured in a flow ramp setting (shear rate from 1 to 2000 (s−1)) and with a 25 mm parallel plate. Using an 8 mm parallel plate, storage (*G*′) and loss (*G*″) moduli of lignin composites were determined by frequency sweeping from 0.62 to 19.9 rad/s at 2% strain. Because storage moduli are altered by axial stress applied during measurement, we evaluated the slope of axial stress vs compression, similar to evaluating Young’s modulus from the slope of a stress–strain curve.^27^ Axial stresses at 0, 10, and 20% of compression were determined, while lignin composites were subjected to 2% strain and 6.28 rad/s frequency.

### 2.7 Animal Model of Wound Healing

Wound healing studies were carried out in wild-type (WT) C57BL/6N mice (8–10 weeks old, female and male). Mice were maintained under pathogen-free conditions with access to food and water *ad libitum* in the Texas Children’s Hospital Feigin Center animal facility. Protocols for animal use were approved by the Institutional Animal Care and Use Committee at Baylor College of Medicine (#AN-6880). At the time of wounding, mice were anesthetized using isoflurane, and the backs were shaved and prepped for surgery with three times alternating betadine and 70% isopropyl alcohol scrubbing. Two 6 mm diameter full thickness wounds were made using a 6 mm dermal punch biopsy, excising tissue through the panniculus carnosus muscle. Skin contraction was controlled through application of a silicone stent with an inner diameter of 8 mm and outer diameter of 16 mm secured concentric to the wound using skin adhesive and six simple interrupted 60 Proline sutures (Ethicon, Raritan, NJ). Wounds were maintained in a moist wound environment using a semiocclusive sterile adhesive dressing (Tegaderm, 3M, St. Paul, MN, USA), and controlling the skin contraction through application of the stent permits wounds to heal in a humanized pattern via granulation tissue deposition and re-epithelialization. Prior to dressing with Tegaderm, each wound received one of the four treatments: a standard saline wash (untreated, UNTX), TLS, CPOc, and CPO (summarized in Table 1). Cross-linking of the treatments was performed immediately after application to the wound bed using a UV flood lamp (B-100AP High Intensity, Blak-Ray) for an exposure time of 30 s. Wounds were imaged at 1, 3, and 7 days postoperatively (Figure S2) and harvested at days 7 and 28. For histology and immunostaining, wounds were bisected in the rostral–caudal plane, fixed overnight in 10% neutral buffered formalin, dehydrated through a series of graded ethanol and xylene, and embedded in paraffin wax. Five-micrometer-thick sections from the paraffin-embedded wounds were collected using an RM 2155 microtome (Leica, Heidelberg, Germany) and used in staining.

### 2.8 Morphometric Quantification

At day 7, epithelial gap and granulation tissue areas were measured from hematoxylin and eosin (cat. #3801571 and #3801615, respectively; Leica, Heidelberg, Germany) stained sections using morphometric image analysis (LASX, Leica, Heidelberg, Germany). Staining was carried out on 5 μm formalin-fixed paraffin-embedded (FFPE) sections following deparaffinization and rehydration in xylene and graded ethanol following the manufacturer’s recommendations. Epithelial gap was determined using a full 4× tile scan of the wound bed and measuring the distance (in mm) between the leading epithelial margins on either side of the wound cross section. The granulation tissue area was determined using a standardized approach of calculating the entire wound area (in mm^2^) above the panniculus carnosus and bounded within the wound edges laterally. If parts of the hydrogel remained devoid of infiltrated cells, those areas were excluded from the granulation tissue assessment.

### 2.9 Immunostaining

Immunohistochemistry was carried out on serial 5 μm sections from day 7 FFPE tissues, which were deparaffinized and rehydrated to water using xylene and graded ethanol. Primary antibodies used were αSMA (rabbit anti-mouse, cat. #ab5694, Abcam, Cambridge, United Kingdom, 1:500 dilution) to measure myofibroblast infiltration, CD31 (rabbit anti-mouse, cat. #ab28364, Abcam, Cambridge, United Kingdom, 1:100 dilution) to measure endothelial cells and vascular lumen density, CD45 (rabbit anti-mouse, cat. #ab10558, Abcam, Cambridge, United Kingdom, 1:5000 dilution) to determine pan-leukocyte infiltration, CD206 (rabbit anti-mouse, cat. #ab64693, Abcam, Cambridge, United Kingdom, 1:500 dilution) to measure M2 macrophage levels, F4/80 (rat anti-mouse, cat. #MF48000, Thermo Fisher Scientific, Waltham, MA, USA, 1:5000 dilution) to measure pan macrophage infiltration, and Ly6G (rat anti-mouse, cat. #551459, BD Biosciences, Franklin Lakes, NJ, USA, 1:5000 dilution) to determine the infiltration of monocytes, granulocytes, and neutrophils.

Following deparrafinization and rehydration to water, sections were then immersed in a target antigen retrieval solution (cat. #K8005212, Agilent, Santa Clara, CA, USA) and treated following the protocol within the DAKO PT Link Rinse Station (cat. #PT10930, Leica, Heidelberg, Germany). Following antigen retrieval, wound sections were stained using the DAKO autostainer (AS480, Leica, Heidelberg, Germany). First, the sections were buffered in DAKO wash buffer (cat. #K800721-2, Agilent, Santa Clara, CA, USA) before incubating in the primary antibody diluted in the DAKO antibody diluent (cat. #S080983-2, Agilent, Santa Clara, CA, USA) for 1 h. The primary antibody was then rinsed, and the sections were washed in a wash buffer before incubation in the appropriate the secondary antibody system, i.e., either HRP-Rabbit (cat. #K400311-2, Agilent, Santa Clara, CA, USA) or HRP-Rat (cat. #D35-110, GBI Labs, Bothell, WA), for 20 min. The secondary antibody was then rinsed off using the wash buffer, and the appropriate visualization system was applied, i.e., DAKO DAB (cat. #K346811-2, Leica, Heidelberg, Germany) for all but CD206 that utilized AEC (cat. # K400511-2, Leica, Heidelberg, Germany). A hematoxylin (cat. #K800821-2, Agilent, Santa Clara, CA, USA) counterstain was applied, sections were dehydrated in xylene and graded ethanol, and coverslips were applied using a xylene-based mounting media. (cat. #23-245691, Thermo Fisher Scientific, Waltham, MA, USA), except for those sections using the AEC visualization system that required an aqueous mounting media (cat. #108562, Merck KGaA, Darmstadt, Germany).

Staining was quantified using images taken on the Leica DMI8 camera. For all data, the percentage of positive cells was determined by counting the number of positive cells per high-powered field (HPF, 40× magnification) and dividing by the total number of cells in that HPF as determined by the hematoxylin counterstain. Final values were determined by the average of four to six HPFs per wound. The total numbers of vessel lumens were counted per HPF. These percent values or vessel counts were then averaged from six images taken across the wound bed to determine the final value.

### 2.10 Scar Assessment

Serial 5 μm sections from D28 FFPE wound sections were deparaffinized and rehydrated to water using xylene and graded ethanol. Sections were then stained using Gomori’s trichrome (blue) following the manufacturer’s recommendations (cat. #38016SS2, Leica, Heidelberg, Germany). The collagen content per HPF in the dermis of the scars was measured using established methods with color-thresholding in ImageJ in which color segmentation was used to isolate only blue pixels, representing collagen fibers, thus allowing the quantification of the amount of collagen within the selected area. Final values were determined by the average of four to six HPFs per wound. Gross images of the wound at day 28 were also obtained, and a subjective assessment of scarring was performed.

### 2.11 Statistical Analysis

For multiple comparisons, one-way ANOVA (analysis of variance) with Tukey’s *post hoc* tests or with the Kruskal–Wallis test followed by Dunn’s test was performed, where *p* values <0.05 or <0.01 were considered significant. At least three independent experiments were performed. Four to five mice were included per treatment per time point.

## 3 Results and Discussion

### 3.1 The Incorporation of CaO_2_ in NPs of SLS-PLGA Led to a Narrower Distribution of NP Diameters

Our effort to apply the antioxidant capability of TLS to wound microenvironments would be synergistic with other proregenerative stimuli. We hypothesized that controlled release of oxygen while scavenging ROS by TLS in the wound microenvironments will promote wound healing. Thus, we incorporated CaO_2_ in NPs of SLS-PLGA/CaO_2_^15^ and found that the size of NPs is not significantly altered even when compared to NPs of SLS-PLGA (w/o CaO_2_), as shown in Table 2. In comparison to SLS, the average diameter of NPs of SLS-PLGA/CaO_2_ increased, whereas the PdI of NPs of SLS-PLGA/CaO_2_ was significantly smaller than that of NPs of SLS-PLGA (w/o CaO_2_). SLS is a biomaterial with potential lot-to-lot variability. However, the synthesis of NP with CaO_2_ slightly increased the average diameter and significantly reduced PdI upon the incorporation of CaO_2_. In contrast, NPs of SLS-PLGA (w/o CaO_2_) were formed only with ethyl acetate, forming irregular shapes possibly with cavity. The average diameter of TLS is significantly smaller than SLS or NPs of SLS-PLGA. After adding TCEP to cleave possible disulfide bonds in TLS, the average diameter of TLS was further reduced. As evidenced in Table 2, TLS formed NPs with a broader distribution. The formation of NPs of SLS-PLGA requires stirring at room temperature, whereas the thiolation of SLS is completed via acid-catalyzed esterification at 80 °C. In PBS (pH 7.4), NPs were formed via interparticle disulfide formation with thiols in TLS, but adding TCEP to PBS dissociates NPs of TLS into smaller NPs. This could be an advantage to form lignin composites with a homogeneous distribution of TLS in the matrix of GelMA.

**Table 2:** Dynamic Light Scattering Results of Nanoparticles^a^

nanoparticles	average dia. ± st. dev. (nm)	PdI_DLS_ (st. dev./mean dia.)^2^
SLS	126.6 ± 7.2	0.585 ± 0.017
SLS-PLGA (w/o CaO_2_)	171.6 ± 1.9	0.204 ± 0.025
SLS-PLGA/CaO_2_	140.8 ± 0.5	0.0597 ± 0.0199
TLS	58.01 ± 13.54	0.258 ± 0.056
TLS in TCEP	7.728 ± 0.090	0.442 ± 0.005

a dia., diameter; st. dev., standard deviation.

**Figure 1:**
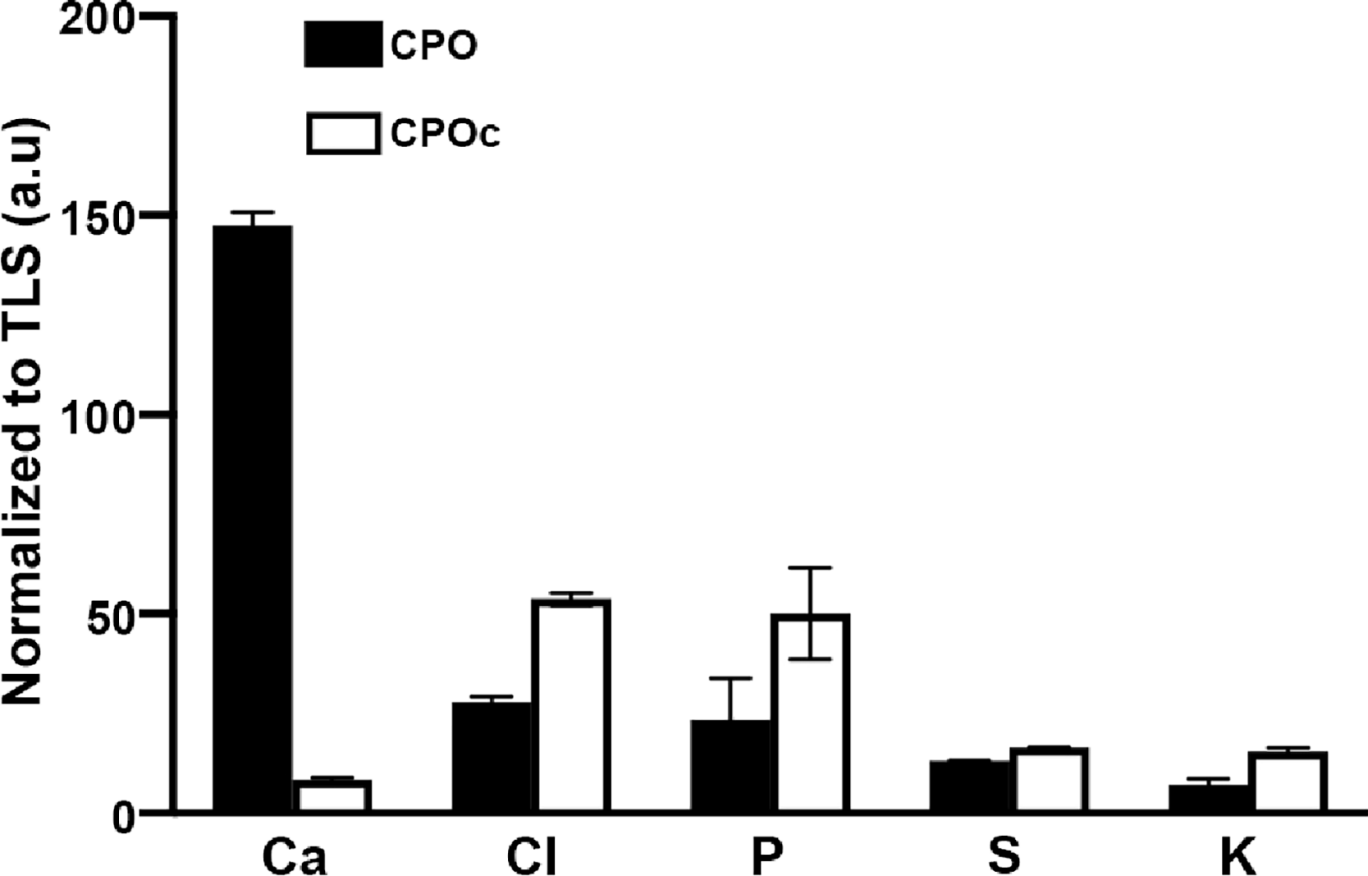
Elemental analysis of NPs of SLS-PLGA with or without CaO_2_ by microPIXE. The concentration (arbitrary units, a.u.) of Ca, Cl, P, S, and K in the NPs normalized to the NPs of TLS. TLS also had trace amounts of Mn and Fe. The bars represent counting uncertainties (1σ) in a single measurement analyzed by the GeoPIXE software; mean ± SEM.

Because of the size (diameter) of NPs, we assessed the incorporation of CaO_2_ by PIXE spectrometry. NPs of SLS-PLGA with or without CaO_2_ showed a similar composition of elements, whereas the normalized concentration of Ca in NPs of SLS-PLGA/CaO_2_ is about 17 times higher than that of NPs of SLS-PLGA (w/o CaO_2_) in the quantitative molecular spectroscopy (Figure 1).

### 3.2 Oxygen Released from CPO Lignin Composites Was Maintained at around 700 ppm/day from Each Composite for 7 Days

**Figure 2:**
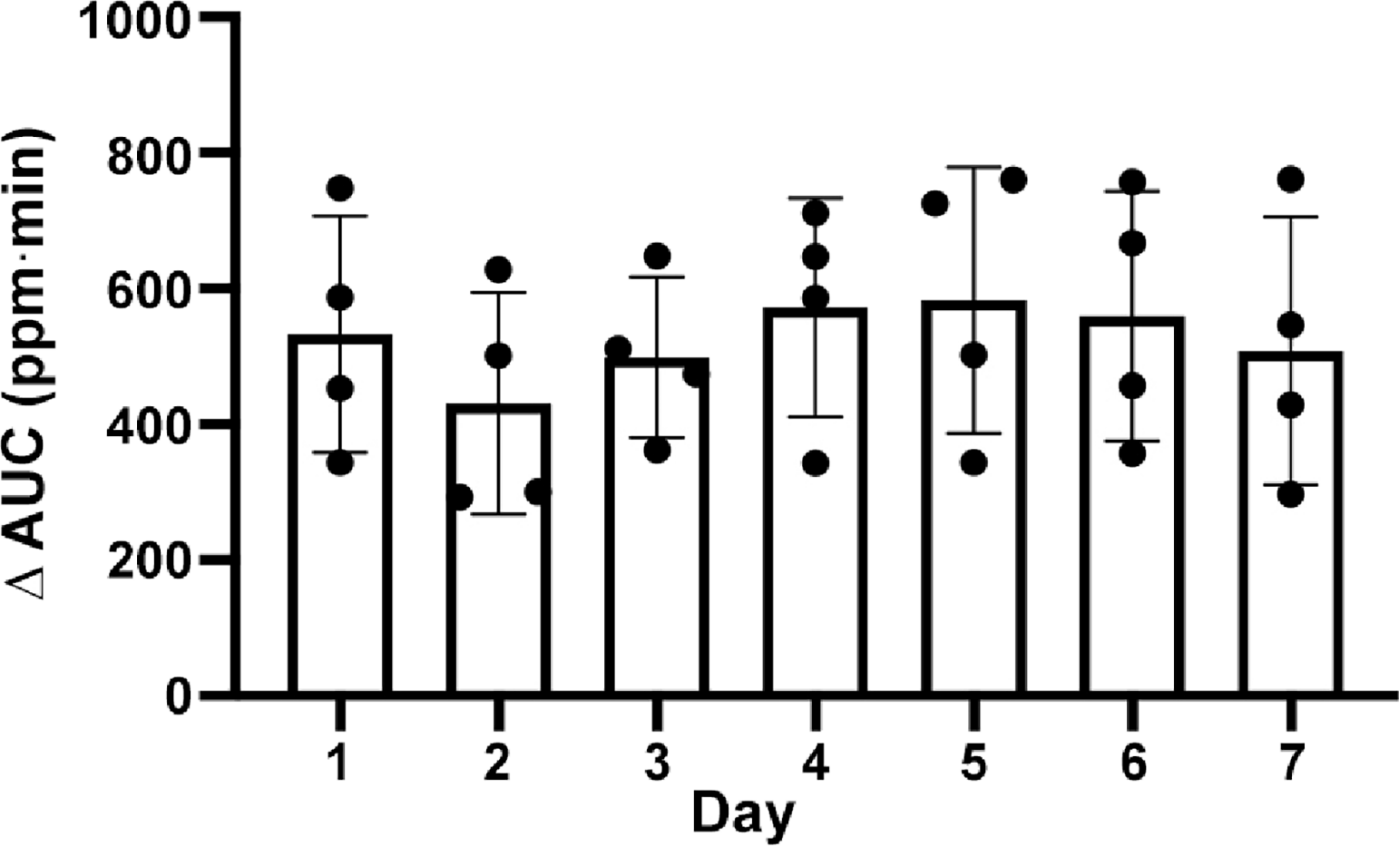
Quantification of O_2_ from lignin composites with NPs of SLS-PLGA/CaO_2_. The area under the curve (AUC) is calculated, and the differential between CPO and CPOc lignin composites over 1440 min is reported. One-way ANOVA with Tukey’s *post hoc* test; no significant difference to each other; mean ± SD, *n* = 4.

To measure the released quantity of O_2_ from lignin composites, we used a planar O_2_ sensor with optical measurement. These planar sensors can measure the concentration of O_2_ in liquid or gas. The difference (Δ) of the area under the curve (AUC) between lignin composites with NPs of SLS-PLGA/CaO_2_ and of SLS-PLGA (w/o CaO_2_) was calculated over 1440 min each day. We also confirmed that the base-level concentration of O_2_ from the GelMA matrix or TLS composite conformed to that of SLS-PLGA (w/o CaO_2_) of around 6.3 ppm (Figure S3). As shown in Figure 2, the difference is around 500 ppm (0.05% O_2_) per day from the lignin composite with 5 mm diameter and 1 mm height (20 μL). This amount can be scaled to 700 ppm per day with lignin composites (6 mm diameter and 1 mm height) for the animal experiments. As the statistical difference is not detected, the oxygen release is maintained up to day 7, which is also distinguished from other methods^28−31^ of O_2_ delivery by CaO_2_. We observed that the swelling of lignin composites over the first 24 h contributed to the slightly higher ΔAUC in day 1 than that in day 2 because lignin composites were placed in a well of the 96-well plate with the planar O_2_ sensor and the serum-free medium was added without achieving equilibrium swelling of lignin composites.

Incorporation of CaO_2_ into NPs of the SLS hydrophilic outer layer and PLGA hydrophobic core^23^ allows CaO_2_ to be complexed with PLGA. When reacted with water at pH lower than 12 (Figure S4A), CaO_2_ can be decomposed into hydrogen peroxide, hydroxide ions, and carbonate, and the generated hydrogen peroxide further decomposes into highly reactive superoxide and hydroxyl radical. Catalase can decompose the intermediate hydrogen peroxide into water and oxygen. Although catalase is an enzyme found in the blood and liver of mammals, this enzyme has to be available *in situ* and not from peroxisome to prevent damages from ROS. This intermediate step eliminates any potential cytotoxic ROS.^32^ Without catalase, the cytotoxic byproduct H_2_O_2_ may lead to cell damage.^33^ Although the actual role of catalase in the oxygen-release process is unclear, decomposition is suggested to take place through the modified Fenton chemistry: dissociation of H_2_O_2_ to OH radicals with Fe^2+^/^3+^.^34^ These oxidants react with everything within the diffusion limit layer, although their half-lives are short. In wound healing, the availability of catalase is limited. Even though the level of expression of catalase mRNA is not changed during wound healing,^35^ the protein level of catalase is decreased.^36^ For example, catalase concentration is reported to be in the range of 50–100 U/mL in the oxygen-generating gelatin section.^37^ In the mixture of CaO_2_, catalase, and either SLS or TLS, the dissociation of H_2_O_2_ is predominantly facilitated by catalase and partly by TLS and to a lesser extent by SLS (Figure S4B). The native antioxidant properties of SLS or TLS NPs (Figure S5A) are maintained at levels of 80% or above of the native antioxidant (l-ascorbic acid) in the presence of up to 50 μg/mL of CaO_2_ (Figure S5B). TLS also showed a ROS scavenging capability in cultures after treating C2C12 myocytes with H_2_O_2_ (Figure S6). We found that the extent of fluorescence intensity from dichlorodihydrofluorescein diacetate (DCFDA) is much diminished in the presence of TLS. Because the complete removal of ROS is also detrimental in wound healing,^38^ the residual ROS detected by the DCFDA assay is indispensable for the survival of C2C12 myocytes. These results implicate that lignin composites effectively scavenge excessive ROS in the earlier phase of wound healing, leading to improved healing of the wounds *in vivo*.

Several cases of CaO_2_ incorporation to gelatin-based biomaterials have been reported to date. A recent study utilized microparticles (MPs) formed with the encapsulation of CaO_2_ in PCL (polycaprolactone). These particles are incorporated in composites with GelMA^39^ and release O_2_ for up to 5 weeks.^40^ Over 2 weeks, 2.5 μM of H_2_O_2_ is released without the formation of MPs, whereas 1.0 μM of H_2_O_2_ is released with the formation of MPs. With 5 to 20 mg/mL of CaO_2_, the cumulative release of O_2_ is 25% without MP and up to 30% with MP. Some cases include catalase (100 U/mL) and CaO_2_ (up to 30 mg/mL).^41,42^ GelMA (50 mg/mL)–CaO_2_ (30 mg/mL) with catalase (100 U/mL) releases O_2_ for up to 5 days.^41^ Another type of hydrogel utilizes Ca^2+^ from CaO_2_ (up to 10 mg/mL) to cross-link gellan gum (anionic polysaccharide) with catalase.^43^ Thiolated gelatin (27–63 mg/mL) with CaO_2_ (25–100 mg/mL) and catalase (2000–5000 U/mg) forms hyperbaric oxygen-generating biomaterials.^44^ The question is how much CaO_2_ is required to efficiently deliver O_2_ to tissue microenvironments without causing cytotoxicity. CaO_2_ concentrations higher than 10 mg/mL exhibit cytotoxicity to 3T3 FBs.^17^ In addition, as a result of the low solubiliuty in water, it is difficult to disperse CaO_2_ in aqueous buffers. Further, Ca^2+^ released from these composites influences both cell cytotoxicity and bacterial adhesion.^45^ Therefore, in our studies, lower CaO_2_ was incorporated in the CPO lignin composites than other gelatin-based, oxygen-generating biomaterials, which resulted in at least 7 days of sustained delivery of O_2._

To delineate the difference, the release kinetics of O_2_ and H_2_O_2_ from CaO_2_ in water with varying pH and temperature values are probed and modeled.^46^ The release of H_2_O_2_ follows a pseudo-zero order reaction. Once CaO_2_ is dissolved in water, Ca^2+^, O_2_^2–^, and H^+^ are formed. Of these, 2H^+^ and O_2_^2–^ form H_2_O_2_. With the increase of H^+^ (decrease of pH), the solubility of CaO_2_ is increased. Temperature has minimal to no effect in the kinetics. In contrast, the release of O_2_ follows a pseudo-first order reaction, meaning that the concentration of CaO_2_ directly affects the dissociation of CaO_2_ to O_2_. With the decrease of pH, the release of O_2_ decreases. With the increase of temperature, the release of O_2_ increases. From this study of kinetics, we surmise that H_2_O_2_ is not necessarily a precursor of O_2_.

### 3.3 Swelling Ratios and Rate of Degradation Were Not Significantly Different with Respect to the Presence or the Concentrations of NPs of SLS-PLGA/CaO_2_

**Figure 3:**
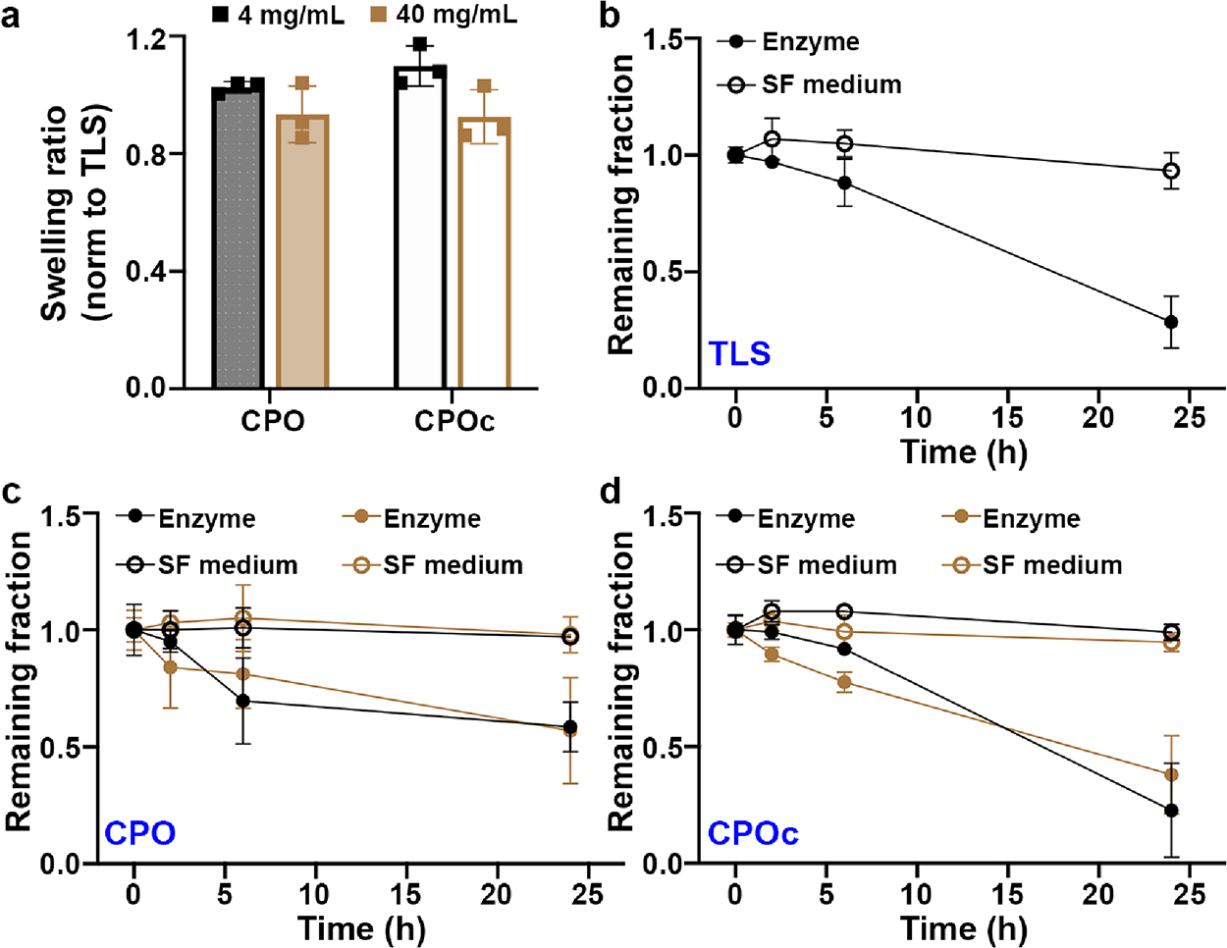
Swelling ratios and degradation of CPO and CPOc lignin composites. (a) Swelling ratios normalized to TLS lignin composites. No statistical difference between CPO and CPOc lignin composites at the same concentration and between two different concentrations at each composite type. Student’s *t* test shows no statistically significant difference; mean ± SD, *n* = 3. Degradation of lignin composites, TLS (b), CPO (c) and CPOc (d), in the solution of 0.5 U/mL collagenase (enzyme) or serum-free medium (SF medium). In panels b–d, black and brown symbols represent the concentrations of NPs (SLS-PLGA with or without CaO_2_) at 4 and 40 mg/mL, respectively; mean ± SD, *n* = 3.

Lignin composites in the wound microenvironment will be subject to swelling and enzymatic degradation and undergo remodeling. Thus, we assessed the extent of swelling of lignin composites by varying the concentration of incorporated NPs of SLS-PLGA with or without CaO_2_. As shown in Figure 3a, the swelling ratios between CPO and CPOc lignin composites were not significantly different at both 4 and 40 mg/mL. With 40 mg/mL of NPs of SLS-PLGA in lignin composites, the swelling ratios were slightly reduced by around 7% (CPO) and 8% (CPOc) without any statistical difference. Apparently, the 10 times higher mass fraction of NPs (regardless of the presence of CaO_2_) in lignin composites contributed to the swelling behavior with marginal difference. To determine the fraction of remaining lignin composites, TLS, CPO (4 and 40 mg/mL), and CPOc (4 and 40 mg/mL) lignin composites were submerged in either collagenase/CaCl_2_/serum-free medium or CaCl_2_/serum-free medium. After 24 h, less than 28% of the TLS lignin composite (Figure 3b), 38% of the CPOc lignin composite with NPs of SLS-PLGA (w/o CaO_2_) at 40 mg/mL, and 23% of the CPOc lignin composite with NPs of SLS-PLGA (w/o CaO_2_) at 4 mg/mL remained (Figure 3d), whereas around 60% of CPO lignin composites remained at both 4 and 40 mg/mL (Figure 3c). All control groups (SF medium in Figure 3b–d) showed minimal (up to 7%) to no changes in the remaining fraction of lignin composites.

The concentration of the collagenase type II used here was 0.5 U/mL (equivalent to 430 μg/mL), which is several orders of magnitude higher than the reported concentrations in patient tissues. For example, the concentrations of matrix metalloproteinases (MMP)-1 and -9 in diabetic foot wounds are estimated to be between 20 and 100 ng/mL.^47^ However, because the concentrations of the MMPs in inflamed tissues vary from patient to patient, the higher collagenase concentration utilized in this study will still cover the ranges that may be encountered *in vivo* under different conditions. Furthermore, native or wounded skin has a plethora of MMPs, their inhibitors, and serum proteins, which lead to a tighter control of degradation, and thus, we expect a slower degradation of lignin composites *in vivo*. Nevertheless, we expect a certain extent of degradation of lignin composites (primarily GelMA) *in vivo* to transiently protect NPs of SLS-PLGA/CaO_2_ from rapid, enzymatic, and mechanical degradation while promoting antioxidant activity from lignosulfonate (both NPs of TLS and SLS in SLS-PLGA).

### 3.4 NPs of SLS-PLGA Did Not Alter the Viscosity of the Lignin Composite Precursors, Whereas the Quantity and Type of NP Modulated the Viscoelasticity of Lignin Composites

**Figure 4:**
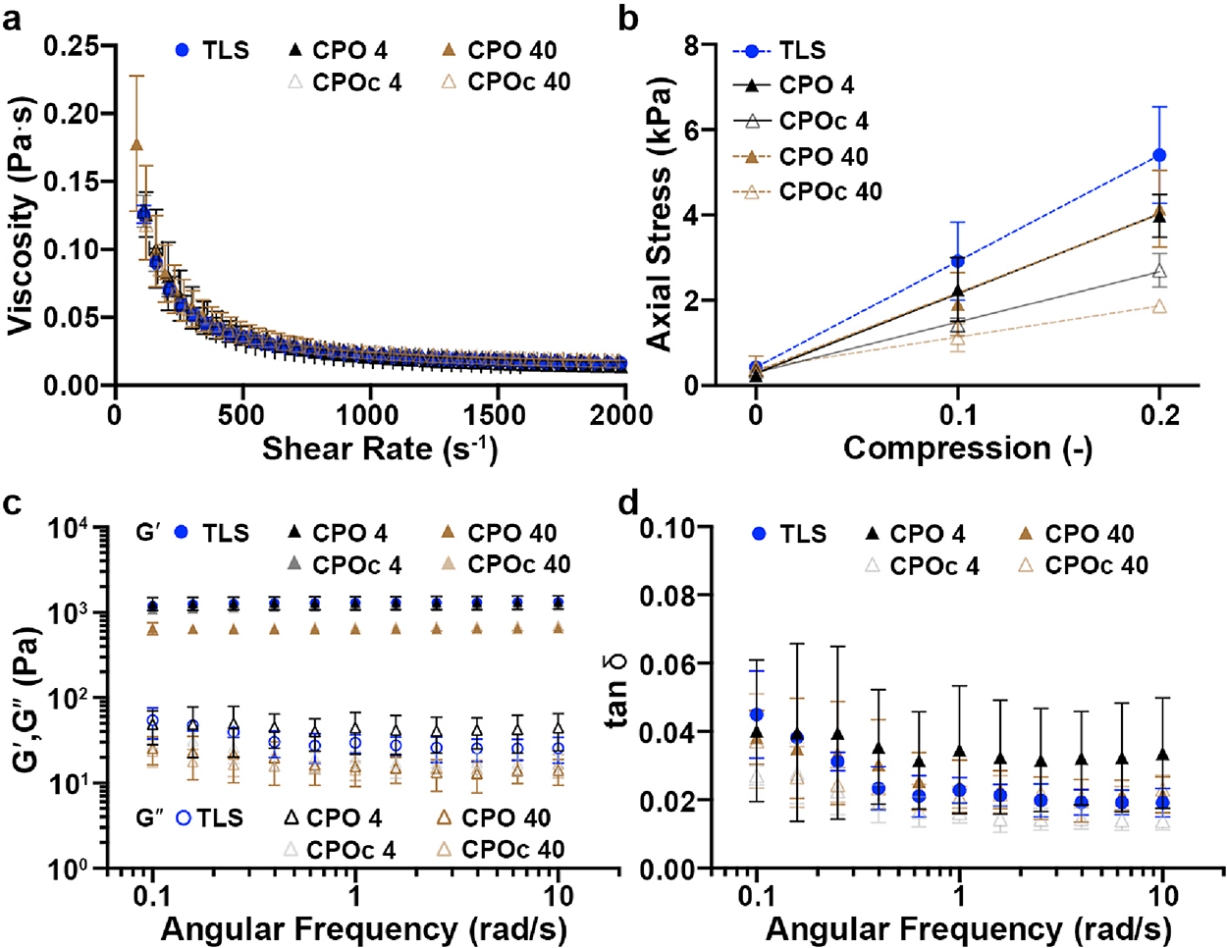
Oscillating rheometry of lignin composites. (a) Viscosity of each precursor before photo-cross-linking. (b) Axial stresses are plotted against compression varying from 0 to 20%. (c) Frequency sweeping of lignin composites. Solid and open symbols represent *G*′ (storage modulus) and *G*″ (loss modulus), respectively. (d) Loss tangent (δ) of lignin composites from 0.1 to 10 rad/s. Mean ± SD, *n* = 3 for all samples.

Because lignin composites are subject to needle injection to the wounded areas, we assessed the mechanical properties of lignin composites before and after thiol-ene cross-linking. The viscosity of all five different types of lignin composite precursors was tested, and we found no significant difference (Figure 4a). Apparently, cross-linked lignin composites exhibited similar viscoelasticity. However, the compressive modulus of elasticity was significantly different upon incorporating NPs of SLS-PLGA/CaO_2_ and SLS-PLGA (w/o CaO_2_). As shown in Figure 4b and Table 3, TLS lignin composites exhibited the highest modulus of elasticity. Upon incorporating NPs of SLS-PLGA/CaO_2_ at 4 or 40 mg/mL, moduli of elasticity were decreased to around 18–19 kPa. While TLS is thiolated SLS to utilize thiol-ene cross-linking,^21^ SLS in NPs of SLS-PLGA was not functionalized for cross-linking. Instead, NPs of SLS-PLGA/CaO_2_ harness CaO_2_ proximal to PLGA chains,^48^ whereas NPs of SLS-PLGA (w/o CaO_2_) were formed without any CaO_2_, which may form cavities during lyophilization. This possibly resulted in lowering the elasticity from the compression test during oscillating rheometry. Consequently, the higher quantity (40 mg/mL) of NPs of SLS-PLGA (w/o CaO_2_) showed much lowered slope down to 7.27 kPa in comparison to that of CPO or TLS lignin composites. In Figure 4c, storage moduli of TLS and CPO lignin composites were similar to each other, whereas those of the CPOc lignin composite were significantly different from those of TLS or CPO lignin composites. However, the loss tangent (*G*″/*G*′) of all lignin composites ranged from 0.01 to 0.07 (equivalent to δ (phase lag) ranging from 0.57 to 4°), indicative of well-cross-linked viscoelastic composites (Figure 4d). Collectively, the reduction of stiffness by non-cross-linkable NPs of SLS-PLGA/CaO_2_ is potentially significant at 40 mg/mL; thus, we continued our investigation of the oxygen-generating capability from lignin composites with NPs of SLS-PLGA (with or without CaO_2_) at 4 mg/mL in mouse models of wound healing.

**Table 3:** Elastic Modulus Estimated by the Slope of Axial Stress vs Compression

lignin composite	slope (elastic modulus, kPa)	intercept (kPa)	*R*^2^
TLS	24.9	0.430	0.8960
CPO 4 mg/mL	18.7	0.286	0.9265
CPO 40 mg/mL	18.4	0.329	0.8661
CPOc 4 mg/mL	11.8	0.304	0.9557
CPOc 40 mg/mL	7.27	0.409	0.9290

### 3.5 The Granulation Tissue Area Was Significantly Increased with CPO Lignin Composites

**Figure 5:**
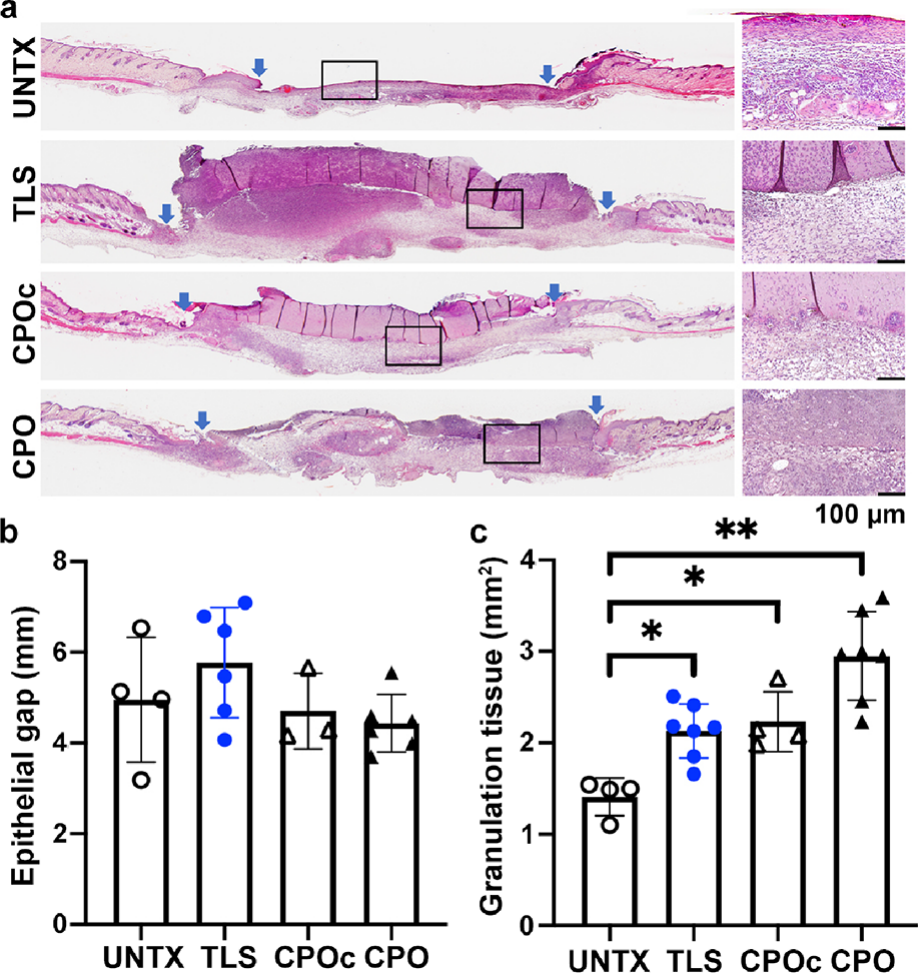
Morphometric analysis of wounds treated with lignin composites at 7 days post wounding. Wounds in WT C57BL/6 N mice were treated with lignin composites (a) to measure the epithelial gap (b) and granulation tissue area (c). In panel a, hematoxylin (blue, nuclei) and eosin (red, ECM and cytoplasm) stained wound sections from different treatments are shown. The left panels show the cross section of the wounds from edge to edge, and the right panels show the corresponding higher magnification of boxed areas (inset) of the granulating wound bed with biomaterial interface. Scale bar, 100 μm. (b) Quantification of the epithelial gap (distance between the blue margins) and granulation tissue area is shown. One-way ANOVA with the Kruskal–Wallis test followed by Dunn’s multiple comparison test was performed. *p < 0.05 and **p < 0.01; 4 ≤ *n* ≤ 7. Bar plots indicate mean ± SD, with individual values from each mouse wound indicated. Details of compositions of UNTX, TLS, CPOc, and CPO are in Table 1.

To determine the effect of lignin composites on the progression of wound healing, wounds were examined on alternate days for the first 7 days post wounding, which represents the early healing stages of inflammation and proliferation. Photographs of the wounds showed that mice tolerated the treatment with different lignin composites well, without any noticeable exudates (Figure S2). Most of the wounds still had the lignin composite hydrogels topically visible even until day 7; thus, assessment of wound closure rate using planimetry to calculate the area of open wounds from the wound pictures was not performed. Wounds were harvested at day 7 post wounding to examine re-epithelialization and granulation tissue formation using H&E staining and morphometric image analysis. Untreated wounds (UNTX in Figure 5a) showed granulation tissue formation and a certain extent of re-epithelialization with encroaching epithelial margins (indicated in blue arrows) with an open wound as expected of the stented murine wild-type wounds at day 7. Wounds treated with TLS or CPOc lignin composites similarly showed granulation tissue formation and a certain extent of re-epithelialization; however, they still showed the separation of the composite matrix from the wound bed tissue (insets in Figure 5a). The CPO lignin composites, in contrast, showed enhanced integration with the granulating wound bed, with cell infiltration uniformly prevalent across the wound cross section. There was no difference noted in the rate of the wound closure, as determined by the epithelial gap (the distance between the encroaching epithelial margins indicated by the arrows), in UNTX and TLS (5.0 ± 1.4 vs 5.8 ± 1.2 mm, *p* > 0.05), CPOc (5.0 ± 1.4 vs 4.7 ± 0.8 mm, *p* > 0.05), and CPO (5.0 ± 1.4 vs 4.4 ± 0.7 mm, *p* > 0.05) lignin composite treated mice (Figure 5b). However, we observed significantly increased granulation tissue area between UNTX and TLS (1.4 ± 0.2 vs 2.1 ± 0.3 mm^2^, *p* = 0.01) or CPOc (1.4 ± 0.2 vs 2.2 ± 0.3 mm^2^, *p* = 0.01) lignin composite treated mice and more in CPO lignin composite treatment (1.4 ± 0.2 vs 3.0 ± 0.5 mm^2^, *p* < 0.01) (Figure 5c), which is indicative of healthy wound-healing progression in all the lignin treated wounds.

### 3.6 CPO Lignin Composites Promoted Wound Neovascularization

**Figure 6:**
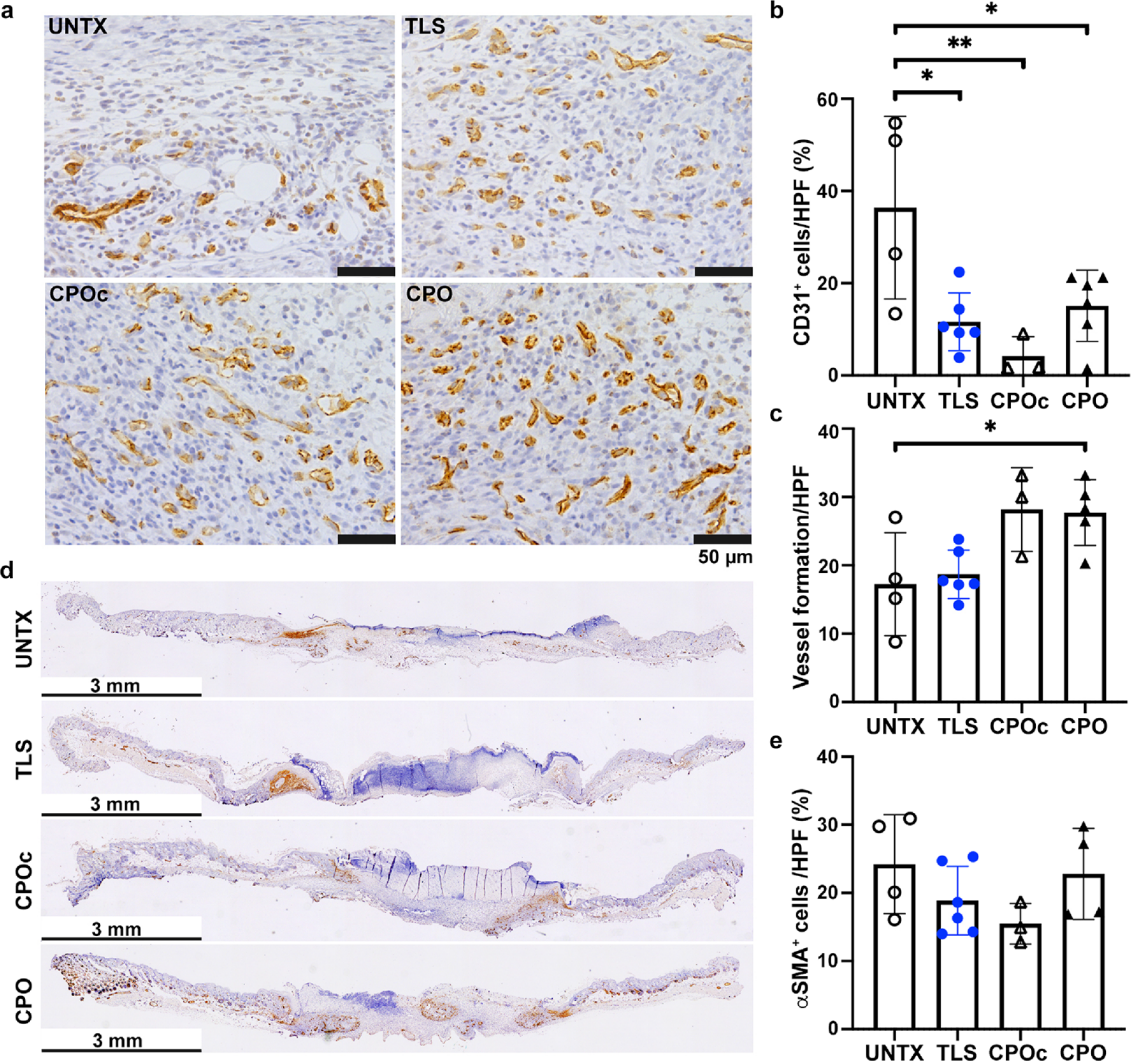
Assessment of neovascularization in the wounds treated with lignin composites at 7 days post wounding. Wounds in WT C57BL/6 N mice were treated with lignin composites (a), and the extent of neovascularization was assessed with immunostaining (CD31, brown) and hematoxylin counterstaining (blue, nuclei). CD31^+^ cells (b) and vessel formation (c) per HPF were quantified. The infiltration of αSMA^+^ cells in the wounds is visualized (d) and quantified per HPF (e). Scale bars, 50 μm in panel a and 3 mm in panel d, respectively. One-way ANOVA with Tukey’s *post hoc* tests, **p < 0.01 and *p < 0.05; 3 ≤ *n* ≤ 6. Bar plots indicate mean ± SD with individual values per each mouse wound indicated. Details of compositions of UNTX, TLS, CPOc, and CPO are in Table 1.

Because neovascularization of the granulating wound bed is a key indicator of wound-healing progression, wound tissue sections were stained with CD31, a marker of ECs. In addition to the increase in granulation tissue formation, neovascularization was also promoted by the CPO lignin composites. CD31 staining revealed that UNTX wounds had a higher number of individual CD31^+^ ECs when compared to lignin composite treatments, whereas the formation of capillary lumens was lower in UNTX wounds at day 7 in these wounds (Figure 6a). Quantification of the CD31^+^ cells per HPF that were not associated with the lumens was first carried out, which showed significantly higher counts per HPF in UNTX wounds than TLS (36.4 ± 19.8 vs 11.6 ± 6.3%, *p* < 0.05), CPOc (36.4 ± 19.8 vs 4.2 ± 4.3%, *p* < 0.01), or CPO (36.4 ± 19.8 vs 15.1 ± 7.7%, *p* < 0.05) lignin composite treated wounds (Figure 6b). Quantification of capillary lumen density per HPF showed significantly fewer lumens in UNTX. However, there were significantly more capillary lumens per HPF in the CPO lignin composite (17.3.2 ± 7.6 vs 17.3 ± 7.5 vessels/HPF, *p* < 0.05) treated wounds as compared to UNTX or TLS wounds (Figure 6c).

In addition, sections were stained for αSMA to detect myofibroblasts in the wounds. As shown in Figure 6d, αSMA^+^ cells (brown staining) were limited to the edge of wounds in UNTX and TLS lignin composites. CPO or CPOc lignin composites showed more αSMA^+^ cells across the whole wound bed. Although the extent of cells at either the edges or middle of the sections was not significantly different among the treatments (Figure S7), the overall trend indicated a slight increase in the αSMA^+^ cells in CPO lignin composites (Figure 6e). Of note, the CPO lignin composite treated wounds revealed a higher number of αSMA^+^ lumens both at the edge and in the middle of wounds (Figure 6d). A review of the literature indicates that αSMA is present in pericytes on capillaries,^49^ and its expression in capillary vessels is associated with the development of vasculature.^50^ The expression of αSMA^+^ by myofibroblasts (myoFB) has also been shown to underlie tissue regeneration in the skin, and the number of αSMA^+^ cells decreases as the regeneration process is completed.^51^ Thus, these data show that neovascularization is promoted and that wound healing is promoted but still in progress by day 7 with the treatment with CPO lignin composite.

### 3.7 Lignin Composites Did Not Cause Significant Inflammatory Responses in the Dermal Wounds

**Figure 7:**
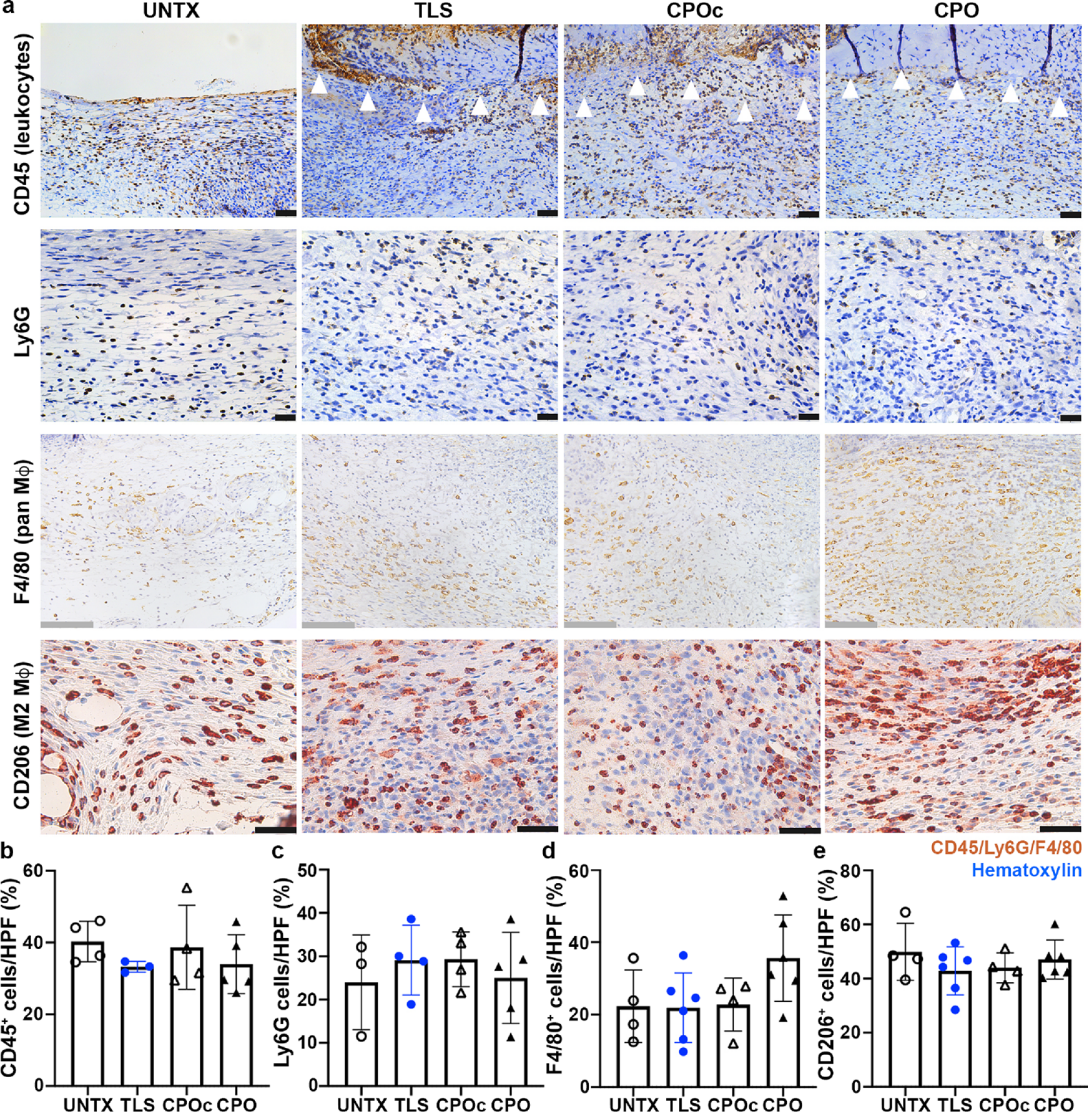
Assessment of inflammatory responses in the wounds treated with lignin composites at 7 days post wounding. Wounds in WT C57BL/6 N mice were treated with lignin composites (a), and the extent of inflammatory responses was assessed with immunohistochemical staining and hematoxylin counterstaining (blue, nuclei). CD45^+^ leukocytes (b); Ly6G^+^ monocytes, granulocytes, and neutrophils (c); F4/80^+^ pan macrophages (d); and CD206 M2 macrophages (e) per HPF are quantified. In panel a, black scale bars: 50 μm and gray scale bars: 125 μm, respectively; 3 ≤ *n* ≤ 6. Bar plots indicate mean ± SD with individual values per mouse wound indicated. Details of compositions of UNTX, TLS, CPOc, and CPO are in Table 1.

Inflammatory responses in the wounds in response to lignin composite treatments were assessed by immunohistochemical staining of wounds sections with a panel of inflammatory markers (Figure 7). CD45 is expressed by common leukocytes except platelet and red blood cells. Ly6G is expressed by monocytes, granulocytes, and neutrophils. F4/80 is used to identify tissue macrophages. CD206 is normally expressed on the alternatively activated, anti-inflammatory (M2) macrophages. At 7 days post wounding, no significant changes in the expression in any the four markers were noted.

As shown in Figure 7a, the interface between wounds and lignin composites does not show (indicated by white wedges) significant infiltration of CD45^+^ cells. There was no significant difference in the percentages of CD45^+^ (pan-leukocyte) cells/HPF across all tested lignin composites when compared to UNTX wounds (UNTX 40.3 ± 5.7 vs TLS 33.3 ± 1.5 vs CPOc 38.7 ± 11.7 vs CPO 34.0 ± 8.2%, *p* > 0.05) (Figure 7b). A similar trend was observed in monocyte, granulocyte, and neutrophil infiltration as determined through Ly6G staining of the wounds (Figure 7c). There were no significant differences in the percentage of Ly6G^+^ cells/HPF among treatment groups (UNTX 24.0 ± 11.0 vs TLS 29.1 ± 8.1 vs CPOc 29.3 ± 6.3 vs CPO 25.1 ± 10.5, *p* > 0.05). No significant differences were observed in macrophages either. F4/80 and CD206 staining (Figure 7c,d) showed that UNTX wounds had no statistically significant differences in the levels of F4/80^+^ cells per HPF (UNTX 22.3 ± 10.0 vs TLS 21.9 ± 9.6 vs CPOc 22.8 ± 7.3 vs CPO 35.6 ± 12.0%, *p* > 0.05) or CD206^+^ cells per HPF (UNTX 49.9 ± 10.5 vs TLS 42.8 ± 8.9 vs CPOc 44.0 ± 5.5 vs CPO 47.0 ± 7.2%, *p* > 0.05).

Recently, the stiffness of GelMA has been shown to direct the macrophage phenotypes. A soft GelMA matrix (stiffness less than 2 kPa) is more favorable for priming macrophages toward M2 phenotypes with a decreased capacity for spreading in comparison to a stiff (stiffness over 10 kPa) GelMA matrix.^52^ The lignin composites used in our wound-healing studies are in the range of 1–2 kPa of storage modulus (Figure 4c), thus likely priming macrophage toward anti-inflammatory (M2) phenotypes. Although the markers to identify the inflammatory cell types, particularly those associated with macrophages, work very well for identifying macrophages using *in vitro* polarization with defined stimuli, there is evidence from the literature that macrophages do not respond to biomaterials in the same way as they do to the biochemical stimuli with distinct polarizations.^53^ For example, Graney *et al.*(54) investigated the behavior of macrophages cultured on ceramic-based scaffolds and found hybrid activation states that were not distinctly M1, M2a, or M2c and further noted that some markers were upregulated whereas others were downregulated in their various scaffold studies, which are not possible to distinguish by cell counting from histologic sections. These studies indicate that increased numbers of phenotype markers are needed to capture the increase in complexity of macrophage phenotypes in biomaterial studies. In this regard, gene expression is being pioneered to characterize macrophage phenotypes more thoroughly.^53^ In particular, alternatively activated M2 macrophages are important mediators of successful wound healing,^55^ and a thorough evaluation of their subtypes in lignin composite wounds would elucidate the mechanisms of their action on promoting wound healing.

In tissue engineering, lignin composites can be applied to enhance mechanical properties with good protein adsorption capacity and wound compatibility,^56^ to confer anti-inflammatory properties by reducing gene expression of inducible nitric oxide synthase (iNOS) and IL-1β of macrophages,^57^ to produce wound dressings with biocompatibility and nontoxicity,^58^ and to achieve enhanced mechanical properties and viability of cells for direct ink writing 3D bioprinting.^59^ Nevertheless, toxicological studies of lignin have only been carried out through simple cytotoxicity testing, which cannot accurately simulate the human body environment. Thus, the biological effects of lignin with animal models should be further tested to assess the long-term stability and potential negative effects of lignin and lignin-derived biomaterials. In addition to toxicological studies, another difficulty or key point for the development of lignin-based biomaterials is the heterogeneity of lignin. Although lignin displays good potential for biomedical applications, its broad distribution of molecular weight and complex structures represent a hurdle for cross-validation deep studies by different groups, standardization, and scale-up. Thus, lignin chemistry and de/polymerization techniques still need to be continuously developed.

### 3.8 Wounds Treated with the CPO Lignin Composite Had Minimal Scar and Exhibited Mature Collagen Architecture at 28 Days post Wounding

**Figure 8:**
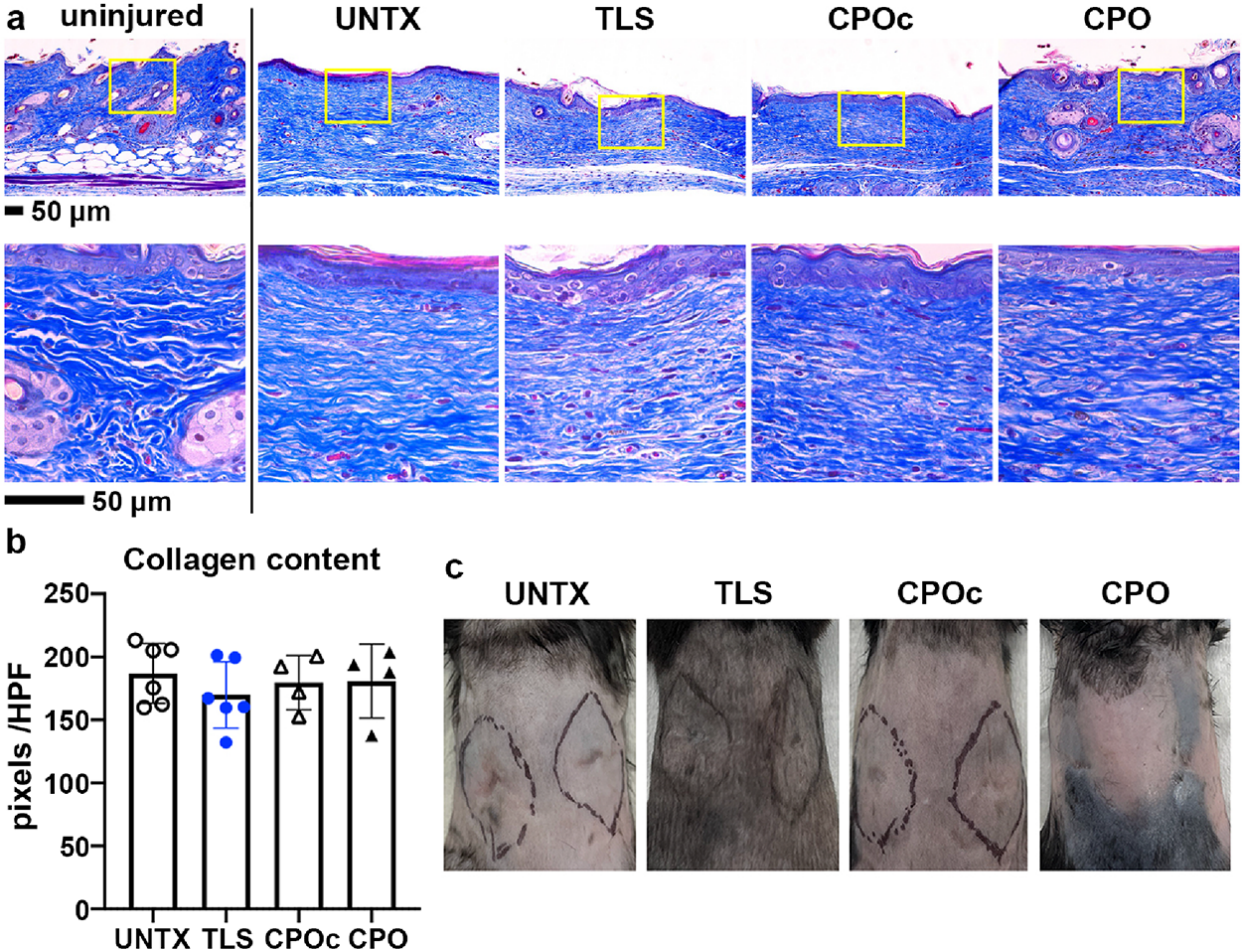
Scar assessment in the wounds treated with lignin composites at 28 days after surgery. Wound sections from WT C57BL/6N mice treated with lignin composites were stained with trichrome (blue, collagen), and the collagen content was assessed. Representative trichrome images of the wounds at low (top row) and high magnification (bottom row) of the area enclosed in yellow boxes are shown (a). Collagen content is quantified per HPF using color thresholding in ImageJ (b). Photographs of wounds at 28 days after surgery. For scar assessment, photographs were taken from all four treatment groups before harvest (c). Scale bar, 50 μm; 3 ≤ *n* ≤ 6. Bar plots indicate mean ± SD with values per mouse wound indicated. Details of compositions of UNTX, TLS, CPOc, and CPO are in Table 1.

As a part of the wound-healing process, murine postnatal skin generally develops scar by 4 weeks post wounding. Therefore, wounds at 4 weeks after treatments were stained with trichrome to examine the dermal architecture and collagen expression. Uninjured “normal” skin has distinct layers of the epidermis. The dermis is composed of the papillary, reticular, and hypodermal subdivisions and also includes the dermal appendages such as hair follicles, sweat gland, and so on. Then, there are a distinct adipose layer and the panniculus carnosus muscle layer in the murine skin. Importantly, the dermal collagen demonstrates a basket-weave pattern in normal skin that renders the skin its stretch and strength. In contrast, the scar tissue that forms after injury lacks dermal appendages, and most often, the adipose and panniculus layers also do not reform. The architecture is also distinct in the scars, with dense ECM and parallel fibers of collagen approximately parallel to the epithelial basement membrane^60^ (Figure 8a). Treatment of the wounds with the CPO lignin composite resulted in a smaller scar compared to untreated or TLS and CPOc treatments. There was a notable reconstitution of dermal appendages in the CPO wounds and traces of the panniculus carnosus muscle layer. The architecture of the collagen also showed a bundled mesh network like a basket weave as opposed to relatively straight fibers found in TLS or CPOc lignin composites (Figure 8a). However, measurements of the collagen content (positive pixels per HPF) did not show significant differences between treatment groups (Figure 8b). No differences were observed in the overall collagen density between UNTX and TLS (187.0 ± 23.8 vs 170.0 ± 26.4 pixels/HPF, *p* > 0.05), CPOc (187.0 ± 23.8 vs 179.6 ± 21.5 pixels/HPF, *p* > 0.05), or CPO (187.0 ± 23.8 vs 180.9 ± 29.4 pixels/HPF, *p* > 0.05) lignin composite treated wounds. These data suggest that the strength of the wounds is not compromised with our treatment groups. Further, wounds treated with the CPO lignin composite left minimal scar as evidenced by photographs taken at 28 days after surgery (Figure 8c). Although we did not see pronounced differences in the collagen content of wounds treated with lignin composites, we were not surprised. Recently, more robust analyses of murine scars have been pioneered by different groups on identifying how closely the collagen fiber density, packaging, presence of dermal appendages, and epidermal topology of the scar resemble those of the normal skin, as these measures are important for the stretch and strength of the repaired skin tissue.^61^ In a study by Mascharak et al.,^61^ a machine learning algorithm was used to quantify the tissue ultrastructure. The latter study utilized Picrosirius Red stained sections, and the images of the wound cross section were color-deconvoluted to isolate ECM fiber components to digitally map thousands of fibers and branch points. Individual (e.g., length, width) and group (e.g., packing, alignment) fiber properties were calculated, and the skin and scars were compared across multiple metrics as opposed to collagen content. However, this is not yet widely available, and qualitative dermatopathological analysis of the histologic sections remains the mainstay. Our future studies can similarly utilize advanced staining and imaging techniques and machine learning to differentiate the types of collagens (i.e., Type I vs Type III), proteoglycans, and other ECM components in the lignin composite wounds. Accordingly, these assessments can further aid in the optimization of the biomaterial properties to accelerate the regeneration of wounds with lignin composites.

Our *in vivo* findings also corroborate our previous work^21^ which showed that the antioxidation capacity of lignin attenuated the expression of fibrotic markers including *COL1A1*, *ACTA2*, *TGFB1*, and *HIF1A* in human dermal fibroblasts (hdFBs). Indeed, we tested different patient-derived fibroblasts from low- to high-scarring phenotypes and showed that lignin composites attenuated the fibrotic markers in high-scar-derived fibroblasts comparable to the phenotype of the low-scar-derived group. To further confirm the attenuation of fibrotic phenotypes of LS (low-scar-derived fibroblast) and HS (high-scar-derived fibroblast) by lignin composites, we analyzed 84 genes associated with angiogenesis, ECM production, and oxidative stress using a fibrosis PCR array. We hypothesized that exposure of LS and HS hdFBs to the TLS lignin composite will attenuate the fibrosis and oxidative stress response genes. As shown in Figure S8, principal component analysis plots showed distinct differences among the HS and LS hdFBs when they were cultured on tissue culture plastic, without any overlap. However, the expression of fibrotic genes in both HS and LS hdFBs on lignin composites was significantly altered, and the HS phenotype appeared to closely associate to that of the LS. Some of the key genes that were altered among the lignin treated and untreated groups included ECM producing genes such as *COL1A1* and *TGFB1* and ECM remodeling genes including *TIMPs* and *MMPs*. These results, together with the previous work,^21^ suggest that addition of lignosulfonate to the wound-healing microenvironment may attenuate fibrotic responses during tissue repair by modulating fibroblast phenotypes. This is an area of prime interest for wound healing, with recent evidence of the involvement of distinct fibroblast lineages in the scar formation processes in wounds.^61^

Although WT mice are programmed to heal physiologically with minimal alterations in perfusion and in the presence of ROS, we did see improvement in our measures of wound healing (Figure 5) and vessel formation (Figure 6) without significant inflammation (Figure 7). Although the quantity of released oxygen from the lignin composites investigated here was around 700 ppm per day *in vitro*, this lignin composite formulation provided an effective dose of oxygen without causing excessive scarring *in vivo*, which is plausible by oxygen-mediated increase in VEGF (vascular endothelial growth factor) stimulation.^62^ Thus, we surmise that the enhanced wound healing by the CPO lignin composite is the product of enhanced vascularization *and* granulation tissue formation. The translatable benefit of the lignin composite may be more readily apparent in disease states such as diabetic wound healing and/or with infection. The advanced glycation end products (AGEs) present in diabetic tissue generate ROS leading to the apoptosis of the FBs via the NLRP3 (NOD-, LRR-, and pyrin domain-containing protein 3) signaling pathway, thus impairing wound repair.^63^ Thus, some of the systemic oral antihyperglycemics currently used to treat diabetes mellitus also have antioxidant and beneficial wound-healing effects. Metformin, for example, is a commonly used medication for glucose control in diabetes mellitus patients that has antioxidant effects and demonstrated benefits on angiogenesis and wound closure in diabetic mice.^64^ However, as previously mentioned, pure systemic antioxidants have a poor enteric absorption profile. Locally applied antioxidant hydrogels have been demonstrated to accelerate diabetic wound healing while promoting M2 macrophage differentiation and reducing IL-1β production.^65^ Further, topical oxygen delivery to ischemic wounds is shown to accelerate healing and promote granulation tissue formation.^66^ Thus, the strategy of combining these dual-functioning, wound-beneficial attributes into one local therapy for pathologic wounds has the potential to greatly benefit the wound healing in at-risk patients.

## 4 Conclusions

Wound-healing applications of lignin have been somewhat limited to doping lignin NPs into a matrix of biomaterials. Here, we functionalized lignosulfonate to form two different types of NPs to scavenge ROS *and* to locoregionally deliver O_2_ without adverse effects on tissue oxygenation. Injection of the CPO lignin composites to wounds resulted in multiple, positive wound-healing responses in that we observed a significant increase in the area of granulation tissue formation and neovascularization as evidenced by significantly increased blood vessel formation and the infiltration of αSMA^+^ cells to the wound by 7 days after surgery. Lignin composites did not cause a significant inflammatory response, and the mechanical properties of the CPO lignin composites were amenable to direct M2-like macrophage phenotype induction in the wound microenvironments. By 28 days, the collagen architecture in the wounds treated with the CPO lignin composite exhibited a more pronounced, bundled basket-weave like network and left minimal scar. Thus, the lignin-based soft matrix with antioxidation (conferred by TLS) and synergistic oxygen release (conferred by SLS-PLGA/CaO_2_ NPs) could be applied to wound-healing applications with enhanced tissue granulation, vascularization, and maturation of collagen architecture without significant inflammatory responses.

## Supporting Information Available

Methods of alkene incorporation in gelatin, cultures of human dermal fibroblasts, real-time transcriptase polymerase chain reaction array, quantification of hydrogen peroxide release from CaO_2_, and assessment of antioxidation capacity of SLS and TLS in the presence of CaO_2_; ^1^H NMR (D_2_O) spectrum of gelatin and GelMA; photographs of wounds after surgery; quantification of αSMA^+^ cells in the wound treated with lignin composites; photographs of wounds at 28 days after surgery; dissociation of peroxide from CaO_2_; antioxidation capacity of SLS and TLS in the presence of CaO_2_; and fibrosis PCR array of human dermal fibroblasts (hdFBs) (PDF)

## Abbreviations

ANOVA: analysis of variance
AUC: area under the curve
DLS: dynamic light scattering
ECs: endothelial cells
ECM: extracellular matrix
FBs: fibroblasts
FFPE: formalin-fixed paraffin-embedded
HPF: high-powered field
hdFBs: human dermal fibroblasts
iNOS: inducible nitric oxide synthase
LAP: lithium phenyl-2,4,6-trimethylbenzoylphosphinate
GelMA: methacrylated gelatin
MPs: microparticles
myoFB: myofibroblasts
NPs: nanoparticles
PIXE: particle-induced X-ray emission
PLGA: poly(lactic-*co*-glycolic) acid
PDMS: polydimethylsiloxane
ROS: reactive oxygen species
SLS: sodium lignosulfonate
TLS: thiolated lignosulfonate
TCEP HCl: tris(2-carboxyethyl)phosphine hydrochloride
WT: wild-type
